# The concomitant use of sodium-glucose co-transporter 2 inhibitors improved the renal outcome of Japanese patients with type 2 diabetes treated with glucagon-like peptide 1 receptor agonists

**DOI:** 10.1097/XCE.0000000000000292

**Published:** 2023-09-28

**Authors:** Kazuo Kobayashi, Masao Toyoda, Nobuo Hatori, Shunichiro Tsukamoto, Moritsugu Kimura, Hiroyuki Sakai, Takayuki Furuki, Keiichi Chin, Tomohiko Kanaoka, Togo Aoyama, Tomoya Umezono, Shun Ito, Daisuke Suzuki, Hiroshi Takeda, Hisakazu Degawa, Toshimasa Hishiki, Hidetoshi Shimura, Shinichi Nakajima, Masaaki Miyauchi, Hareaki Yamamoto, Yutaka Hatori, Masahiro Hayashi, Kazuyoshi Sato, Masaaki Miyakawa, Yasuo Terauchi, Kouichi Tamura, Akira Kanamori

**Affiliations:** a Committee of Hypertension and Kidney Disease, Kanagawa Physicians Association; b Department of Medical Science and Cardiorenal Medicine, Yokohama City University Graduate School of Medicine, Yokohama; c Division of Nephrology, Endocrinology and Metabolism, Department of Internal Medicine, Tokai University School of Medicine, lsehara; d Division of Nephrology, Department of internal medicine, Kitasato University School of Medicine, Sagamihara; e Department of Endocrinology and Metabolism, Yokohama City University Graduate School of Medicine, Yokohama, Japan

**Keywords:** glucagon, like peptide 1 receptor agonist, renal outcome, sodium, glucose co-transporter 2 inhibitors

## Abstract

**Aims:**

This study aimed to clarify the renal influence of glucagon-like peptide 1 receptor agonists (GLP1Ras) with or without sodium-glucose co-transporter 2 inhibitors (SGLT2is) on Japanese patients with type 2 diabetes mellitus (T2DM).

**Methods:**

We retrospectively extracted 547 patients with T2DM who visited the clinics of members of Kanagawa Physicians Association. The progression of albuminuria status and/or a ≥ 15% decrease in the estimated glomerular filtration rate (eGFR) per year was set as the renal composite outcome. Propensity score matching was performed to compare GLP1Ra-treated patients with and without SGLT2i.

**Results:**

After matching, 186 patients in each group were compared. There was no significant difference of the incidence of the renal composite outcomes (17% vs. 20%, *P* = 0.50); however, the annual decrease in the eGFR was significantly smaller and the decrease in the urine albumin-to-creatinine ratio was larger in GLP1Ra-treated patients with the concomitant use of SGLT2is than in those without it (−1.1 ± 5.0 vs. −2.8 ± 5.1 mL/min/1.73 m^2^, *P* = 0.001; and −0.08 ± 0.61 vs. 0.05 ± 0.52, *P* = 0.03, respectively).

**Conclusion:**

The concomitant use of SGLT2i with GLP1Ra improved the annual decrease in the eGFR and the urine albumin-to-creatinine ratio in Japanese patients with T2DM.

## Introduction

Several CVOTs using sodium-glucose co-transporter inhibitors (SGLT2is) have demonstrated the significant superiority of these agents to placebo in not only cardiovascular outcomes [1–3] but also renal outcomes [1–4]. Furthermore, dapagliflozin and empagliflozin showed superiority to placebo with regard to renal outcomes in patients with chronic kidney disease with or without diabetes mellitus (DM) [5,6]. Based on such robust evidence concerning SGLT2is, their use is now increasing in clinical practice. Dipeptidyl peptidase-4 inhibitor (DPP4i) and Glucagon-like peptide 1 receptor agonist (GLP1Ra) are incretin-related hypoglycemic agents that stimulates insulin secretion on β cells. According to the 2019 national report by the Ministry of Health, Labour and Welfare, DPP4i is the most frequently prescribed drug in Japanese patients with type 2 DM (T2DM) due to its efficacy in decreasing the plasma glucose level and relatively low frequency of adverse effects [7]. However, DPP4is have shown only non-inferiority to placebo with regard to major adverse cardiac outcomes in several cardiovascular outcome trials (CVOTs) [8–11]; further, concerns remain regarding the increased heart failure associated with the use of saxagliptin [10]. In contrast, GLP1Ras showed their superiority to placebo with regard to cardiovascular outcomes by CVOTs [12–15]; however, evidence of their superiority concerning renal outcomes is limited to CVOTs including GLP1Ras [12,16]. Based on the above evidence, the use of GLP1Ras is also widespread in Japan, although the extent of their expansion is less than that of SGLT2is [7].

Furthermore, most CVOTs using GLP1Ras were performed mainly in the USA and Europe, and the cardiovascular or renal events by GLP1Ras in Asian patients with T2DM have not been adequately studied. In addition, in Japan, liraglutide was only available at doses lower than the world standard when it was released, and only low dose of dulaglutide is currently used. Therefore, whether the results of CVOTs using GLP1Ras can be applied to Japanese patients with T2DM are not enough discussed.

Our retrospective survey showed the renoprotective effects of SGLT2is in clinical practice in Kanagawa Prefecture, Japan; however, the effects of long-term GLP1Ra usage on the renal function in clinical practice have not yet been sufficiently discussed or reported. Therefore, we also conducted the retrospective survey to evaluate the influence of GLP1Ras on the renal function in Japanese patients with T2DM. In our survey, approximately half of the included patients were treated with SGLT2i and GLP1Ra in combination, therefore, the analysis that considers the concomitant use of SGLT2i which has already been shown to be renoprotective was needed.

Hence, the present study aimed to clarify the influence of GLP1Ras on the renal function with or without SGLT2i on Japanese patients with T2DM.

## Materials and methods

This study was approved by the special ethics committee of the Kanagawa Medical Association, Japan (approval Krec202005 on 23 March 2020 for the GLP1Ra survey and this comparison survey, and approval Krec304401 on March 6, 2018, for the SGLT2i survey).

### Study subjects and data collection

We conducted a retrospective survey of patients with T2DM using GLP1Ra agents. A schematic illustration of the study design is provided in Supplementary Figure S1, Supplemental digital content 1, http://links.lww.com/CAEN/A43. Kanagawa Physicians Association, that consisted of approximately 1600 general physicians in Kanagawa prefecture, conducted this retrospective study. We invited all members to participate in this survey and asked to collect the data of the study subjects who visited their clinics between July and October 2020 and matched to the inclusion criteria as follows; study subjects were (i) patients with T2DM, and (ii) treated with GLP1Ras between July and October 2020, (iii) treated with GLP1Ra continuously for more than 1 year, and (iv) ≥20 years old in 2022. The exclusion criteria were (i) type 1 DM; (ii) requirement for chronic dialysis; (iii) severe liver dysfunction (e.g. liver cirrhosis or severe infection), (iv) terminal-stage malignancy, (v) pregnancy, (vi) irregular use of GLP1Ras (suggested by poor adherence), and (vii) intent to opt out during the study.

Twenty-two medical facilities participated in this study, and totally the data of 637 patients were collected. Based on these criteria, 34 patients were excluded. The median and average duration of GLP1Ra treatment was 47 and 52 months, respectively (range, 12–126). Therefore, in the case of the patients who have used GLP1Ra for the longest time, the data from 2009 was included in this study as baseline data. To evaluate renal outcomes, the following parameters were recorded, both at the time of the initiation of GLP1Ra treatment and at the time of the survey: age, sex, height, BW, BP [systolic BP (SBP), diastolic BP (DBP)], serum creatinine level, glycated hemoglobin A1c (HbA1c) level, and results of urinary tests (urine albumin-to-creatinine ratio [ACR] [mg/g Cr] or qualitative proteinuria). The estimated glomerular filtration rate (eGFR) was calculated using the following formula: eGFR (mL/min/1.73 m^2^) = 194 × age^-0.287^ × serum creatinine^-1.094^ × (0.739 for women) [1]. Qualitative proteinuria values were converted to albuminuria values using the formula reported by Sumida *et al*. [2]. Due to missing ACR data at the time of initiation or during the survey, 56 patients in the GLP1Ra survey were excluded, with 547 GLP1Ra-treated patients ultimately included.

Among all included GLP1Ra-treated patients, 282 with the concomitant use of SGLT2i and 265 without it were compared to assess the influence of the concomitant use of SGLT2is on the renal outcomes.

### BP measurements at the office

BP measurements’ method that was utilized in this study were written in our previous report [3]. Office BP measurements were measured at each institution using their own validated cuff oscillometric devices. According to the JSH 2014 guidelines [4] (11), general practitioners measured office BP in a quiet environment after resting for a few minutes in the seated position on a chair with the legs uncrossed.

### Outcomes

To determine the influence of the concomitant use of SGLT2is, the progression of the ACR status and/or a ≥ 15% decrease in eGFR per year was set as the renal composite outcome, as with our previous reports [5,6]. Second, the change in clinical findings included the logarithmic value of the ACR (ΔLnACR) and the annual change in the eGFR (annualΔeGFR) after GLP1Ra treatment were evaluated.

### Statistical analyses

Data that showed a normal distribution were reported as the mean±SD, while those that showed a skewed distribution were reported as the median (25th percentile, 75th percentile). The IBM SPSS Statistics 28.0 software program (IBM Inc., Armonk, NY, USA) was used for the statistical analyses, and *P* values <0.05 were considered significant.

Adjusting for factors that might influence the outcome (confounding factors) is necessary for a retrospective cohort survey to evaluate the differences in the renal composite outcomes. In the present study, we performed a propensity score (PS) analysis to balance the confounding factors between the two groups. PSs for GLP1Ra-treated patients administered SGLT2is were calculated using a logistic regression model with the following variables: age, sex, BW, HbA1c, SBP, DBP, and eGFR and LnACR at baseline, as well as the concomitant use of other glucose-lowering agents and statins.

To compare the difference in the effects of GLP1Ra in patients with the concomitant use of SGLT2i and without it, we performed a PS matching analysis to adjust the confounding factors at baseline. In this study, PS for the concomitant use of SGLT2i was calculated using logistic regression analysis. PS matching was done at the initiation of GLP1Ra treatment. The algorithm of PS matching was a 1:1 nearest neighbor match with a caliper value of 0.040, calculated as 0.2-fold of the SD of PS [7] with no replacement. Comparisons between two groups for the clinical characteristics were performed using an unpaired *t*-test for the parametric variables, the Mann–Whitney rank-sum test for non-parametric variables, and the chi-square test for the categorical data in the unmatched cohort model. The paired *t*-test for parametric variables, Wilcoxon’s signed-rank test for non-parametric variables, and McNemar’s test for categorical data were used in the PS-matched cohort model.

## Results

### Changes in the clinical characteristics after GLP1Ra treatment

In this retrospective study, the median duration of GLP1Ra treatment was 47 (26–68) months. The clinical backgrounds of the 547 patients at the initiation of GLP1Ra treatment are shown in Table 1. Concomitant medications, including glucose-lowering drugs, antihypertensive drugs, and statins, at the time of the survey are also listed in Table 1.

**Table 1:** Clinical backgrounds of the 547 patients assessed at the initiation of GLP1Ra treatment

Age (years)	63.3 ± 13.0 (range; 27–89)
Gender (Male) (n [%])	301(55%)
The duration of the history of T2DM; <10/≥10 years/unknown (n [%])	13 (2.4%)/162 (29.6%)/372 (68.0%)
BW (kg)	76.0 ± 19.5
BMI (kg/m^2^)	28.5 ± 5.9
SBP/DBP/MAP in the office (mmHg)	132.1 ± 17.0/75.8 ± 12.1/94.6 ± 12.0
SBP/DBP/MAP at home (mmHg) (n = 95)	127.2 ± 13.1/73.4 ± 9.1/91.3 ± 8.7
HbA1c (% [mmol/mol])	8.4 ± 1.6 (68 ± 17)
Cr (mg/dL)	0.88 ± 0.47
eGFR (mL/min/1.73 m^2^)	73.7 ± 26.4
ACR (mg/gCr) [25th–75th percentiles]	28.0 [11.5–110.6]
LnACR	1.61 ± 0.77
≤30/>30 to <300/≥300 (mg/g Cr, n [%])	286 (52%)/175 (32%)/86 (16%)
The types of GLP1Ra	
Liraglutide	224 (41%)
Dulaglutide	186 (34%)
Exenatide	14 (3%)
Lixisenatide	9 (2%)
Change in GLP1Ra during the administration period in 114 cases.	
Duration of administration of GLP1Ra [25^th^-75^th^ percentiles] (month)	47 [26, 68]
Concomitant drugs at the survey	
Glucose lowering drugs	Average 2.0 drugs (maximum: 6)
GLP1Ra alone	109 (20%)
DPP4i	5 (1%)
Sulphonylurea	121 (22%)
Metformin	277 (51%)
Insulin	228 (42%)
SGLT2 inhibitor	282 (52%)
Pioglitazone	87 (16%)
Others (αGI, glinides, etc.)	84 (15%)
Antihypertensive drugs	Average 1.4 drugs (maximum: 6)
none	217 (40%)
RAS inhibitor	308 (56%)
Ca channel blocker	243 (44%)
Aldosterone blocker	30 (6%)
Diuretic (thiazides)	47 (8%)
Diuretic (lupus)	35 (6%)
β-blocker	80 (15%)
Others (α-blockers, etc.)	21 (4%)
Other	
Statins	355 (65%)

Values are expressed as the mean ± SD, median [25th-75th percentiles], or n (n/total [%]).

BW, body weight; DBP, diastolic blood pressure; DPP-4, dipeptidyl peptidase-4; GLP1Ra, glucagon-like peptide-1receptor agonist; LnACR, logarithmic value of urine albumin-to-creatinine ratio; MAP, mean arterial pressure; RAS, renin-angiotensin system; SBP, systolic blood pressure; SGLT2, sodium-glucose co-transporter 2; T2DM, type 2 diabetes mellitus; αGI, alpha-glucosidase inhibitor.

Table 2 shows a comparison of the clinical findings in the 574 patients at the time of the initiation of GLP1Ra treatment and at the survey. GLP1Ra treatment did not significantly change the median ACR value, which increased only slightly from 28.0 (11.5–110.6) to 28.7 (10.9–120.6) mg/g Cr, but the eGFR significantly decreased from 73.7 ± 26.4 to 67.1 ± 26.8 mL/min/1.73 m^2^ (*P* < 0.001). The HbA1c, BW, and SBP/DBP/MAP at the office also decreased significantly (*P* < 0.001) after GLP1Ra treatment (all *P*-values were <0.001).

**Table 2:** Comparison of the clinical findings of the 547 patients at the initiation of GLP1Ra treatment and at the survey

	At the initiation of GLP1Ra treatment	At the survey	*P* value
ACR (mg/gCr) [25th-75th percentiles]	28.0 [11.5, 110.6]	28.7 [10.9,120.6]	0.13
Ln ACR	1.61 ± 0.77	1.63 ± 0.79	0.54
eGFR (mL/min/1.73 m^2^)	73.7 ± 26.4	67.1 ± 26.8	*P* < 0.001
HbA1c (% [mmol/mol])	8.4 ± 1.6 (68 ± 17)	7.6 ± 1.3 (60 ± 14)	*P* < 0.001
BW (kg)	76.0 ± 19.5	73.6 ± 19.5	*P* < 0.001
SBP in the office (mmHg)	132.1 ± 17.0	129.3 ± 15.7	*P* < 0.001
DBP in the office (mmHg)	75.8 ± 12.1	73.8 ± 11.8	*P* < 0.001
MAP in the office (mmHg)	94.6 ± 12.0	92.3 ± 11.5	<0.001

Values are expressed as the mean ± SD or median [25th–75th percentiles].

BW, body weight; DBP, diastolic blood pressure; DPP-4, dipeptidyl peptidase-4; GLP1Ra, glucagon-like peptide-1receptor agonist; LnACR, logarithmic value of urine albumin-to-creatinine ratio; MAP, mean arterial pressure; RAS, renin-angiotensin system; SBP, systolic blood pressure; SGLT2, sodium-glucose co-transporter 2; T2DM, type 2 diabetes mellitus; αGI, alpha-glucosidase inhibitor.

### PS-matched cohort model

Two groups that consisted of 186 PS-matched patients were compared. Table 3 and supplementary Figure S2, Supplemental digital content 2, http://links.lww.com/CAEN/A44 shows the clinical characteristics at baseline and the distribution of PS for the PS-unmatched and PS-matched cohort models. The largest standardized difference in background covariates in the PS-matched cohort model with 186 patients in each group was 0.08, showing no significant difference between the two groups and confirming that the PS-matched model was well-balanced.

**Table 3:** Clinical characteristics at the initiation of GLP1Ra treatment in patients with and without the concomitant use of SGLT2is in the unmatched and matched cohort models

	Unmatched cohort (n = 547)		*P*-value	Matched cohort (n = 372)			
	SGLT2i (-) (n = 265)	SGLT2i (+) (n = 282)		SGLT2i (-) (n = 186)	SGLT2i (+) (n = 186)	*P*-value	Standardized difference
Age (years)	66.2 ± 13.3	60.5 ± 12.1	<0.001	63.4 ± 13.4	63.4 ± 11.4	0.91	0.00
Gender (male)	132 (50%)	169 (60%)	0.02^a^	99 (53%)	101 (54%)	0.91^b^	0.02
>10 years of DM	88 (33%)	74 (26%)	0.08^a^	53 (29%)	50 (27%)	0.82^b^	0.04
BMI (kg/m^2^)	27.4 ± 5.5	29.6 ± 6.1	<0.001	28.1 ± 5.6	28.2 ± 5.1	0.84	0.02
BW (kg)	71.5 ± 17.4	80.2 ± 20.5	<0.001	74.2 ± 18.1	74.8 ± 16.5	0.69	0.04
SBP (mmHg)	131.5 ± 16.0	132.8 ± 18.0	0.37	132.2 ± 16.1	131.9 ± 18.0	0.85	0.02
DBP (mmHg)	74.2 ± 11.3	77.4 ± 12.6	0.002	75.3 ± 11.2	75.7 ± 12.4	0.76	0.03
MAP (mmHg)	93.3 ± 11.1	95.8 ± 12.7	0.01	94.3 ± 11.2	94.4 ± 12.6	0.92	0.01
HbA1c (% [mmol/mol])	8.3 ± 1.6 (68 ± 18)	8.5 ± 1.6 (69 ± 17)	0.34	8.4 ± 1.6 (69 ± 17)	8.3 ± 1.5 (68 ± 16)	0.62	0.05
eGFR (mL/min/1.73 m^2^)	70.2 ± 25.3	77.0 ± 27.1	0.002	74.0 ± 24.6	73.9 ± 26.9	0.94	0.004
LnACR	1.59 ± 0.75	1.64 ± 0.79	0.48	1.61 ± 0.80	1.61 ± 0.80	0.98	0.00
ACR	27.1[11.5, 96.8]	30.9[11.1, 125.6]	0.51	26.5[11.1, 109.1]	29.3[10.7, 109.6]	0.80	
Duration of administration of GLP1Ra (month)	54.7 ± 31.3	49.5 ± 29.6	0.04	52.6 ± 30.1	51.8 ± 29.9	0.80	0.03
The types of GLP1Ra							
Liraglutide	119 (45%)	105 (37%)	0.07^a^	76 (41%)	81 (43%)	0.65^b^	0.05
Dulaglutide	84 (32%)	102 (36%)	0.27^a^	63 (34%)	59 (32%)	0.74^b^	0.05
Exenatide	9 (3%)	5 (2%)	0.23^a^	5 (3%)	5 (3%)	1.00^b^	0.00
Lixisenatide	4 (2%)	5 (2%)	0.81^a^	4 (2%)	5 (3%)	1.00^b^	0.03
Change in GLP1Ra^¶^	49 (19%)	65 (23%)	0.19^a^	38 (20%)	36 (19%)	0.90^b^	0.03
Concomitant treatment at the survey							
Metformin	109 (41%)	168 (60%)	<0.001^a^	95 (51%)	96 (52%)	1.00^b^	0.01
Sulphonylurea	46 (17%)	75 (57%)	0.009^a^	38 (20%)	33 (18%)	0.58^b^	0.07
Insulin	115 (43%)	113 (40%)	0.43^a^	84 (45%)	88 (47%)	0.77^b^	0.04
Pioglitazone	36 (14%)	51 (18%)	0.15^a^	31 (17%)	29 (16%)	0.88^b^	0.03
RAS inhibitors	147 (56%)	161 (57%)	0.70^a^	112 (60%)	107 (58%)	0.67^b^	0.05
Ca channel blocker	124 (53%)	119 (58%)	0.28^a^	90(48%)	83 (45%)	0.53^b^	0.08
Β blocker	38 (14%)	42 (15%)	0.86^a^	28 (15%)	30 (16%)	0.89^b^	0.03
Statins	159 (60%)	196 (70%)	0.02^a^	121 (65%)	123 (66%)	0.91^b^	0.02

Values are expressed as the mean ± SD or n (n/total [%]), and the analysis was performed using an unpaired *t*-test or chi-square test

a in the unmatched model and a paired *t*-test or McNemar’s test

b in the matched model.

BW, body weight; DBP, diastolic blood pressure; DPP-4, dipeptidyl peptidase-4; GLP1Ra, glucagon-like peptide-1receptor agonist; LnACR, logarithmic value of urine albumin-to-creatinine ratio; MAP, mean arterial pressure; RAS, renin-angiotensin system; SBP, systolic blood pressure; SGLT2, sodium-glucose co-transporter 2; T2DM, type 2 diabetes mellitus; αGI, alpha-glucosidase inhibitor.

### The comparison of renal composite outcomes

The incidence of the renal outcomes and the clinical characteristics after GLP1Ra treatment in the matched cohort model are shown in Table 4. No significant differences were observed in the renal composite outcomes; however, the annual decrease in the eGFR was significantly smaller and the decrease in the LnACR larger in the GLP1Ra-treated patients with the concomitant use of SGLT2i than in those without it (−1.1 ± 5.0 vs. −2.8 ± 5.1 mL/min/1.73 m^2^, *P* = 0.001; and −0.08 ± 0.61 vs. 0.05 ± 0.52, *P* = 0.03, respectively). The changes in the BW, SBP, DBP, and MAP after GLP1Ra treatment did not show significant differences between the two groups.

**Table 4:** Renal outcomes and clinical characteristics after GLP1Ra treatment in the matched cohort model

	SGLT2i (-) n = 186	SGLT2i (+) n = 186	*P*-value
Renal outcomes and the function			
a) Incidence of renal composite outcome	38 (20%)	32 (17%)	0.50
progression of ACR worsening	29 (16%)	26 (14%)	0.77
≥15% decrease in the eGFR per year	10 (5%)	6 (3%)	0.45
b) ΔeGFR			
Change rate of the eGFR (%)	−13.6 ± 19.5	−5.6 ± 21.0	<0.001^a^
Annual change in the eGFR (mL/min/1.73 m^2^/year)	−2.8 ± 5.1	−1.1 ± 5.0	0.001^a^
c) Changes in the LnACR	0.05 ± 0.52	−0.08 ± 0.61	0.03
Clinical characteristics after GLP1Ra treatment			
eGFR (mL/min/1.73 m^2^)	64.0 ± 23.7	69.5 ± 26.6	0.03^a^
LnACR	1.66 ± 0.84	1.53 ± 0.76	0.14^a^
BW (kg)	71.9 ± 17.8	72.1 ± 16.4	0.89^a^
SBP (mmHg)	130.9 ± 15.6	128.3 ± 15.9	0.10^a^
DBP (mmHg)	73.7 ± 12.3	73.2 ± 11.1	0.68^a^
MAP (mmHg)	92.7 ± 11.7	91.5 ± 11.3	0.30^a^
HbA1c (% [mmol/mol])	7.6 ± 1.3 (59 ± 14)	7.6 ± 1.2 (60 ± 13)	0.68^a^

Values are expressed as the mean ± SD or n (n/total [%]), and the analysis was performed using a paired *t*-test or McNemar’s test

a in the matched model.

BW, body weight; DBP, diastolic blood pressure; DPP-4, dipeptidyl peptidase-4; GLP1Ra, glucagon-like peptide-1receptor agonist; LnACR, logarithmic value of urine albumin-to-creatinine ratio; MAP, mean arterial pressure; RAS, renin-angiotensin system; SBP, systolic blood pressure; SGLT2, sodium-glucose co-transporter 2; T2DM, type 2 diabetes mellitus; αGI, alpha-glucosidase inhibitor.

## Discussion

In the present study, we retrospectively collected a relatively large population of Japanese patients with T2DM (n = 547) to whom GLP1Ras had been continuously prescribed for long periods in clinical practice (median treatment period: 47 months). Furthermore, as in our previous study of SGLT2i-treated patients [8], this study included detailed clinical findings, unlike other database studies using only the International Statistical Classification of Diseases and Related Health Problems-10 (ICD10) code or medical and pharmaceutical claims. To our knowledge, no research on GLP1Ras like the present study has previously been conducted.

As GLP1Ras treatment decreased not only the glucose level but also the BW or BP, potential renoprotective effects were speculated; however, any marked improvement in the ACR or decrease in the eGFR was not observed in total GLP1Ra-treated patients in this study. In contrast, on the PS-matched model in this study, there was no significant difference of the incidence of the renal composite outcomes (17% in GLP1Ra-treated patients with SGLT2i vs. 20% in those with it, *P* = 0.50), however, GLP1Ra-treated patients with the concomitant administration of SGLT2i showed renoprotective effects, including a smaller decrease in the annual ΔeGFR and larger decrease in the LnACR than GLP1Ra-treated patients without SGLT2i (−1.1 ± 5.0 vs. −2.8 ± 5.1 mL/min/1.73 m^2^, *P* = 0.001; and −0.08 ± 0.61 vs. 0.05 ± 0.52, *P* = 0.03, respectively).

### GLP1 and the decrease in ACR

Albuminuria is the most important surrogate marker for the progression of DM nephropathy [9] and an independent factor for not only end-stage kidney disease but also cardiovascular events [10]. Several experimental models have demonstrated the renoprotective effects of GLP1Ras without lowering the blood glucose level [11]. In clinical practice, a small study of 23 patients receiving liraglutide treatment for 1 year showed a 27% decrease in the mean ACR (from 25.5 to 18.6 mg/day) [12]. In addition, a small study of 31 patients receiving 1.8 mg/day liraglutide demonstrated a 7-mmHg reduction in the 24-h SBP (*P* = 0.11) and reduction in the ACR by 30% (95% confidence interval (CI): 12–44%, *P* = 0.003) [13].

Regarding GLP1Ras and SGLT2is, several CVOTs were performed to evaluate the cardiovascular outcomes. The LEADER trial with liraglutide [14], SUSTAIN-6 trial with semaglutide [15], REWIND trial with dulaglutide [16], HARMONEY trial with albiglutide [17], and AMPLITUDE-O trial with efpeglenatide [18] demonstrated the superiority of the respective agents to placebo with regard to major adverse cardiovascular events, with hazard ratios (95% CI) of 0.87 (0.78–0.97), 0.74 (0.58–0.95), 0.88 (0.79–0.99), 0.78 (0.68–0.90), and 0.73 (0.58–0.92), respectively.

The renal outcomes were additionally analyzed as a secondary outcome in the LEADER trial, SUSTAIN-6 trials, REWIND trial, and AMPLITUDE-O trial. These outcomes included the progression of the ACR, decrease in the eGFR, induction of kidney replacement therapy, and renal death, and different studies defined renal outcomes slightly differently (e.g. progression to microalbuminuria or macroalbuminuria for the ACR outcome and a 30% or 40% decrease in the eGFR for the GFR outcome). Therefore, care should be taken when interpreting the renal outcome findings in each trial. Kristensen *et al*. conducted a meta-analysis for the renal composite outcome with GLP1Ra treatment, and GLP1Ra treatment showed superiority to placebo with regard to the renal composite outcomes, including progression to macroalbuminuria 0.83 [95% CI, 0.78–0.89], but no such effects were seen in those without GLP1Ra treatment 0.87 [95% CI, 0.73–1.03] [19]. Importantly, a superior renal outcome in these trials was seen not in the prevention of the deterioration of the renal function but in the inhibition of the progression of overt albuminuria, except for in the EXSCEL trial with weekly exenatide using specific statistical analyses and adjustments [20]. Details concerning the renal outcomes and function after long-term liraglutide treatment are shown in the LEADER trial [14] [21]. After 36 months, in the liraglutide group, the ACR and eGFR changed from 21 to 22.8 mg/gCr and from 80.2 to 72.8 mL/min/1.73 m^2^, respectively, while in placebo group, the ACR and eGFR changed from 21 to 27.3 mg/gCr and from 80.6 to 70.8 mL/min/1.73 m^2^. The AMPLITUDE-0 trial also showed a similar change in the ACR, going from 28.3 to 53 mg/gCr in patients with efpeglenatide treatment and from 28.3 to 64 mg/gCr in patients administered a placebo [18]. To summarize the results of CVOTs using GLP1Ras, the ACR worsened slowly in the placebo group over several years of observation; however, GLP1Ra treatment reduced the ACR by 10–20% compared to the placebo group.

The changes in the ACR and eGFR in all 574 GLP1Ra-treated patients in this study were +0.7 mg/gCr and −6.6 mL/min/1.73 m^2^ at a median of 47 months. Our study included other GLP1Ras as well and when limited to liraglutide-treated patients (n = 224), the ACR and eGFR changed from 28.0 (11.0–184.4) to 28.9 (11.5–160.5) mg/gCr (*P* = 0.37) and from 73.0 ± 26.1 to 65.6 ± 26.3 mL/min/1.73 m^2^, respectively (*P* < 0.001), which may be similar to the findings in the liraglutide-treated patients in the LEADER trial. Our finding of no marked change in the ACR (i.e. it did not worsen) may suggest that GLP1Ra treatment prevented the progression of ACR worsening. Further, it is interesting that the GLP1Ra treatment tend to decrease the LnACR from 1.61 ± 0.80 at baseline to 1.53 ± 0.76 at the survey only in patients with the concomitant use of SGLT2i (*P* = 0.07).

### The combination of GLP1 and SGLT2i

Another important point to consider when interpreting the results of CVOTs using GLP1Ras is the concomitant use of SGLT2is. Several CVOTs using SGLT2is have demonstrated the significant superiority of these agents to placebo in not only cardiovascular outcomes but also renal outcomes [22–26]

Because most patients enrolled in CVOTs have a high risk of cardiovascular disease or end-stage kidney disease (ESKD), the proportion of patients which the concomitant use of SGLT2is during the study periods increased. Indeed, in the LEADER trial, 2.1% of patients in the liraglutide group and 2.8% of patients in the placebo group received an SGLT2i during the study period [14]. In the EXSCEL trial, the ratio of the concomitant use of an SGLT2i was 1.2% in the exenatide group and 0.7% in the placebo group at baseline, increasing to 6.5% and 9.4%, respectively, during the study period [27]. In the HARMONEY outcome trial, the ratio was 7% in the albiglutide group and 6% in the placebo group at baseline [17], and in the PIONEER-6 trial, the ratio was 10.4% in the semaglutide group and 8.8% in the placebo group at the last observation time [28].

In the AMPLITUDE-0 trial, the most recently published study, SGLT2is were already being used in 15% of patients in each group at baseline [18]. The type of SGLT2i, concomitant timing, administration period, dosage, etc. did not necessarily match between the GLP1Ra group and placebo group; therefore, the possibility that SGLT2is affected the outcome cannot be denied. Indeed, in the present study, a multiple regression analysis identified the use of an SGLT2i as an independent factor both for the improvement of the LnACR and the annual ΔeGFR. Furthermore, because 52% of patients in this study were treated with GLP1Ras and SGLT2is, it was necessary to compare the renal outcomes between the groups with and without the concomitant use of SGLT2is.

GLP1Ra treatment with concomitant SGLT2i administration showed superiority to GLP1Ra treatment without concomitant SGLT2i administration with regard to annual ΔeGFR and changes in the LnACR. Based on these results, the concomitant use of an SGLT2i achieved renoprotective effects, particularly an improvement in the decrease in the eGFR during GLP1Ra treatment.

Because GLP1Ras mainly act renoprotective by inhibiting the progression of ACR worsening, these renoprotective effects may be mediated by SGLT2is. The concomitant use of an SGLT2i also resulted in a larger decrease in the LnACR than without one. In this PS-matched model, the ACR changed from 26.5 (11.1–109.1) to 26.0 (10.2–139.2) in GLP1Ra-treated patients without concomitant SGLT2i administration. However, whether or not GLP1Ra treatment without an SGLT2i worsens the ACR still needs to be determined by a comparison with a control group.

### Study limitations

Several limitations associated with the present study warrant mention. The present study was a retrospective observational study that included only patients who could be continuously treated with GLP1Ras. Therefore, patients who discontinued GLP1Ra treatment for any reason were excluded, so selection bias could not be avoided. Because short-acting GLP1Ras delay gastric emptying, adverse effects, such as loss of appetite or abdominal bloating, are thought to be more common than with other hypoglycemic drugs. Furthermore, all GLP1Ras that were surveyed in this study were administered by injection, so poor adherence or an impaired quality of life may be major concerns compared to oral hypoglycemic drugs. Given these concerns, an intent-to-treat analysis may lead to more realistic results than an on-treatment analysis in observational studies.

Next study limitation is the data on the concomitant use of SGLT2i. During the survey period from July to October 2020, six types of SGLT2i were available for patients with DM to control the plasma glucose level in Japan (In November 2020, Dapagliflozin was approved for the use to patients with chronic heart failure in Japan.) In this study, we did not survey the reason of the concomitant use of SGLT2is. In many cases, SGLT2is were used for improving the glycemic control, however, SGLT2is sometimes might be used for switching other hypoglycemic drugs. Further, we did not survey the data of the timing of the initiation of SGLT2i treatment, therefore, this analysis included two types of patients; patients who were treated with GLP1Ra and later, SGLT2i was added, and patients who were already treated with SGLT2i and later, GLP1Ra was initiated. In addition, the data of the administration period of SGLT2i were not collected. This study focused on whether SGLT2i was used as concomitant drug or not in GLP1Ra-treated patients and it is not a complete data analysis of the combination treatment. Therefore, a study including data of the initiation time and the administration period of both drugs is needed to analyze the influence of the combination therapy of SGLT2i and GLP1Ra more accurately.

Third, PS matching is useful for balancing the confounding factors at baseline; however, not all confounding factors can be balanced. The influence of uninvestigated or unknown confounding factors therefore cannot be denied. Furthermore, after PS-matching, the sample size was reduced by nearly half, which led to poor statistical power.

Finally, some significant differences were observed in this study, however, After the initiation of GLP1Ra, the clinical data was collected only at the time of final observation. Data collection were not performed during the study. Therefore, we could not evaluate when the difference occurs in this study.

Anti-metabolic effects by GLP1Ra, such as lowering BP, HbA1c, and BW will be associated with the improvements in MACE, however, unfortunately, we have no data for MACE. Future study is needed for the evaluation of MACE, further, larger sample sizes and longer observational periods may be necessary for it.

### Conclusion

In this retrospective study including Japanese T2DM patients with T2DM who were treated with GLP1Ras, these agents failed to exert substantial renoprotective effects; however, the concomitant use of SGLT2is with GLP1Ras improved the annual decrease in the eGFR and the ACR.

## Acknowledgements

We are grateful to all participants and acknowledge the support of Fuyuki Minagawa, Hideo Machimura, Shinichi Umezawa, Noriko Kanayama, Nobumichi Saito, Kouta Aoyama, Masahiro Takihata, Kohsuke Minamisawa, Yoshiro Hamada, Sanae Takeichi, Yoshiro Suzuki, Mitsuo Obana, Atsuko Mokubo, Noriyuki Asaba, and Satoshi Suzuki, who contributed considerably to data collection.

Data are available from the Kanagawa Physicians Association Data Access/Ethics Committee for investigators, bound by confidentiality agreements.

### Conflicts of interest

There are no conflicts of interest.

## Supplementary Material


