# Supplementary material for: The concomitant use of sodium-glucose co-transporter 2 inhibitors improved the renal outcome of Japanese patients with type 2 diabetes treated with glucagon-like peptide 1 receptor agonists
Source: Cardiovasc Endocrinol Metab. 2023 Sep 28;12(4):e0292. doi: 10.1097/XCE.0000000000000292 (PMC10540913; doi:10.1097/XCE.0000000000000292)
Supplement: Supplementary file 1 [file xce-12-e0292-s001.pdf]

Supplementary Figure S1. Schematic illustration of the study design

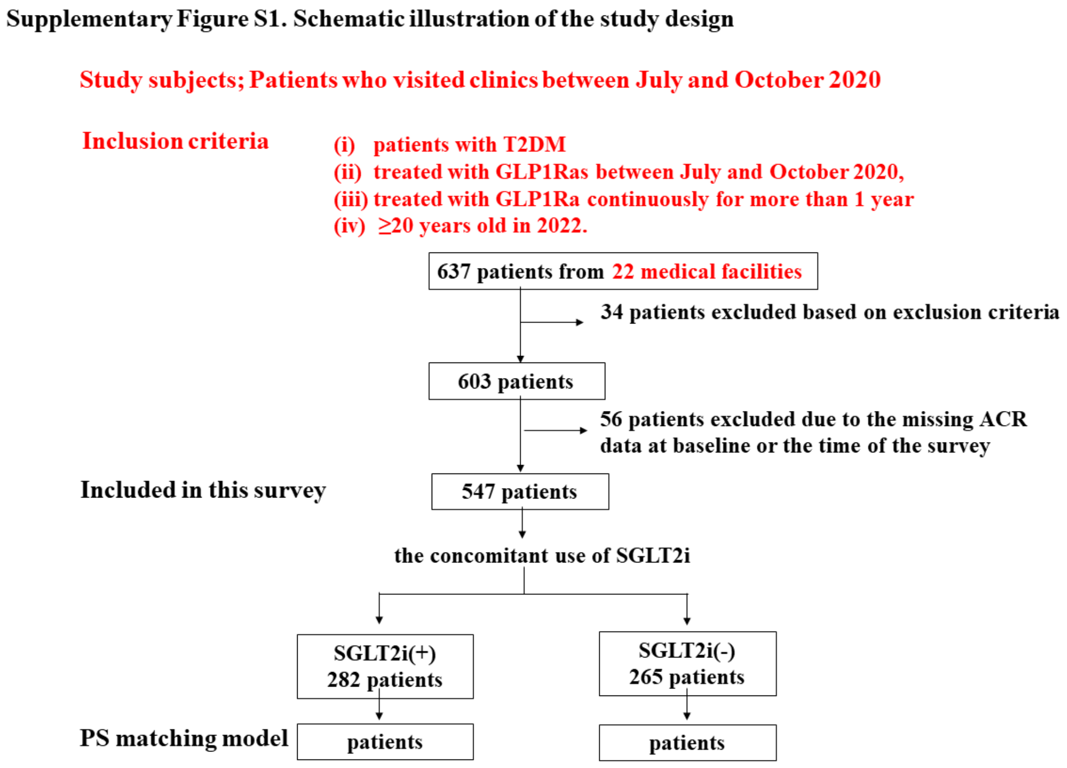

GLP1Ra, glucagon-like peptide 1 receptor agonist; PS, propensity score; SGLT2i, sodium-glucose co-transporter inhibitor
